# Trust Index Based Fault Tolerant Multiple Event Localization Algorithm for WSNs

**DOI:** 10.3390/s110706555

**Published:** 2011-06-27

**Authors:** Xianghua Xu, Xueyong Gao, Jian Wan, Naixue Xiong

**Affiliations:** 1 Grid and Services Computing Lab, School of Computer Science and Technology, Hangzhou Dianzi University, Hangzhou 310037, China; E-Mails: huxy20@yahoo.com.cn (X.G.); wanjian@hdu.edu.cn (J.W.); 2 Department of Computer Science, IS&T on Virtual Computing Lab, Georgia State University, Atlanta, GA 30303, USA; E-Mail: xiongnaixue@gmail.com

**Keywords:** trust index, binary data, multiple event localization, fault tolerance, maximum likelihood estimation, wireless sensor networks

## Abstract

This paper investigates the use of wireless sensor networks for multiple event source localization using binary information from the sensor nodes. The events could continually emit signals whose strength is attenuated inversely proportional to the distance from the source. In this context, faults occur due to various reasons and are manifested when a node reports a wrong decision. In order to reduce the impact of node faults on the accuracy of multiple event localization, we introduce a trust index model to evaluate the fidelity of information which the nodes report and use in the event detection process, and propose the Trust Index based Subtract on Negative Add on Positive (TISNAP) localization algorithm, which reduces the impact of faulty nodes on the event localization by decreasing their trust index, to improve the accuracy of event localization and performance of fault tolerance for multiple event source localization. The algorithm includes three phases: first, the sink identifies the cluster nodes to determine the number of events occurred in the entire region by analyzing the binary data reported by all nodes; then, it constructs the likelihood matrix related to the cluster nodes and estimates the location of all events according to the alarmed status and trust index of the nodes around the cluster nodes. Finally, the sink updates the trust index of all nodes according to the fidelity of their information in the previous reporting cycle. The algorithm improves the accuracy of localization and performance of fault tolerance in multiple event source localization. The experiment results show that when the probability of node fault is close to 50%, the algorithm can still accurately determine the number of the events and have better accuracy of localization compared with other algorithms.

## 1. Introduction

Wireless Sensor Networks (WSNs) consist of many sensor nodes capable of computation and communication which are distributed in a specified area. The sensor nodes can collaborate to deal with many kinds of complicated tasks including monitoring ecological environments, protecting infrastructures, tracking targets and so on [1–3]. WSNs which are deployed in a real environment may easily fail due to many reasons, such as software malfunctions, hardware failures, radio interference, battery depletion, malicious damage and so on [4–6]. As mentioned in [5], about 40% to 60% of data measured by sensor nodes can be faulty in a real environment deployment. Therefore, fault-tolerance is a particular important issue in WSN applications.

WSNs are often used to detect the occurrence of an event in a region and determine its location, such as monitoring of pollution sources, detection of fire occurrence and so on. In these applications, all events are continually emitting signals whose strength is attenuated inversely proportional to the distance from the source. The sensor nodes report the strength of the signal to the sink regularly, and then the sink estimates the location of the source according to the information of the alarmed nodes reporting. The event localization algorithms can be divided into centralized approaches and distributed approaches. In a centralized approach, all sensor measurements are sent to the sink, and the location estimation is performed at the sink [7–9]. In a distributed approach, nodes exchange sensors observation information with the surrounding neighbors and determine who is the cluster node [10–12]. The cluster nodes run a localization algorithm and determine the location of the sources. Centralized approaches can collect more information and accurately determine the location of the events, but they always consume more energy. Distributed approaches, on the other hand, have less computation overhead, but are not accurate enough for determining the location of the events. This paper mainly focuses on the fault-tolerance issue for multiple event detection and localization in wireless sensor networks, and devises a simple, fault-tolerant multiple event localization algorithm with higher estimation accuracy.

Maximum likelihood estimation is an important approach used for event localization [13–16]. Michaelides [17] proposed a distributed multiple event source localization algorithm based on maximum likelihood estimation. In the algorithm, each node exchanges information with the surrounding neighbors and some nodes are elected as cluster nodes. Then, the cluster nodes construct the likelihood matrix by analyzing the information of its neighbor nodes. Finally, the cluster nodes determine the location of all the events through maximum likelihood estimation. However, when constructing the likelihood matrix, faulty nodes may have a great effect on the value of the maximum likelihood matrix elements and result in a great deviation of positioning.

In this paper, we introduce the *trust index* for each sensor node, which used to evaluate the trust degree of a node according to its previous alarm reporting and determine the weight of the node’s reporting data in the event localization process, to reduce the impact of faulty nodes in event localization. We propose the Trust Index based Subtract on Negative Add on Positive (TISNAP) localization algorithm, which reduces the impact of faulty nodes on the event localization by decreasing their trust index, to improve the accuracy of event localization and performance of fault tolerance for multiple event source localization. The algorithm has three main phases: determine the number of events, localization and updating of the trust index: (1) the sink identifies the cluster nodes to determine the number of events occurred in the entire region by analyzing the binary data reported by all nodes. First, the alarmed nodes send binary data to the sink and other nodes remain silent. Next, the sink computes all the likelihood functions *F_n_* according to the collected data. Each alarmed node *n* has a corresponding likelihood function *F_n_*. If *F_n_* > 0, we think that there is an event around the alarmed node *n*. Then the alarmed node whose corresponding likelihood function value is the maximal value in a certain area is selected as a cluster node; (2) the sink constructs the likelihood matrix related to the cluster nodes and estimates the location of all events according to the alarmed status and trust index of the nodes around the cluster nodes; (3) the sink updates the trust index of all nodes according to the behavior in the previous reporting. According to the location of all nodes and their reported data, the sink judges whether or not the data reported by them is true. If it is judged true, the sink increases the trust index of the node. Otherwise, the sink reduces its trust index. The trust index of nodes ranges from 0 to 1. By introducing the trust index model, the algorithm enhances the influence of normal nodes and reduces the influence of faulty nodes, and it has higher localization accuracy and better performance of fault tolerance.

The paper is organized as follows: first, in Section 2, we present the related work in event localization in sensor networks. Next, in Section 3, we introduce the model we have adopted and the underlying assumptions. In Section 4, we provide the details of the TISNAP algorithm for multiple event source localization. In Section 5, we theoretically compare the TISNAP algorithm with the DSNAP algorithm. Section 6 presents the simulation results and comparison of the performance with other algorithm. Finally, in Section 7, we present the conclusions of our research.

## 2. Related Works

Event localization is an important research issue in WSNs [13,14]. The localization techniques can be classified into four main categories: (1) Angle of Arrival (AOA) [18]; (2) Time of Arrival (TOA) [19,20]; (3) Time Difference of Arrival (TDOA) [21,22]; (4) Energy-based [9,11,23,24]. The energy-based approach uses event signal strength of sensor measurements to estimate event location [13–17]. It does not need precise synchronization among the sensor nodes. Hence, it is more suitable for event localization in large scale wireless sensor networks.

Ding proposed the Centroid Estimator (CE) algorithm [9]. It first gets the middle value of the sampling, filtering the incorrect data caused by occasional faults. Then it simply takes the centroid of the positions of all alarmed sensor nodes as the estimated event location. Let (*x_n_*, *y_n_*), *n* = 1, 2, …, *P* (*p* ≤ *N*) denote the position of all alarmed sensor nodes. Then, the event location estimated by CE is the centroid of these positions:

(1) $${\hat{\theta }}_{CE}=[{\hat{x}}_{s},{\hat{y}}_{s}]=[\frac{1}{P}\sum_{n=1}^{P}x_{n},\frac{1}{P}\sum_{n=1}^{P}y_{n}]$$

However, this algorithm is sensitive to the presence of false positives (sensor nodes not in the region of the source but alarmed). These faults can result in large errors, especially if the faulty node is far away from the event location.

Niu [15] proposed an algorithm called Maximum Likelihood (ML) that uses only binary readings which are communicated to the base station to estimate the event position. The likelihood function is given by:

(2) $$\text{log}(p|\theta )=\sum_{n=1}^{N}\sum_{m=1}^{M}I_{n,t}\times \text{log}[Q(\frac{T-S_{n}(\theta )}{\sigma_{\omega }})]+(1-I_{n,t})\times \text{log}[1-Q(\frac{T-S_{n}(\theta )}{\sigma_{\omega }})]$$

where *I_n,t_* is the binary reading. *S_n_*(*θ*) is the measured signal by sensor without any noise. ML is sensitive to false negatives (nodes detected the event but not alarmed). These faults can result in large errors, especially for the faulty nodes close to the event that do not become alarmed.

Michealidis proposed Subtract on Negative Add on Positive (SNAP) [16] for event location only using binary data from the sensor nodes. The main idea is that the base station uses the binary observations to construct a matrix by adding ±1. The size of the matrix is fixed and the sensor is at the center of the area. Specifically speaking, the alarmed sensors add 1 to the region of their coverage, while the silent sensors subtract 1. By summing the contribution of each sensor, the maximum of the matrix points to the estimated event location. The Add on Positive (AP) algorithm is a variant of the SNAP algorithm. It only uses positive contributions from the alarmed sensors to construct the likelihood matrix. It may be used to obtain a low-complexity implementation and can be robust to false negatives, but it has low accuracy.

Sheng [8] presented a maximum likelihood (ML) acoustic source localization method which use the intensity attenuation function of acoustic signal. Analog measurements from sensors are required to estimate the source location. This incurs high communication and computation overhead. Therefore, it is desirable that only binary or multi-bit data are transmitted from local sensors to the processing node in the context of resource limited WSNs.

In the DSNAP [17] algorithm and SNAP [16] algorithm, binary data from local sensors is transmitted to the sink to estimate the location of events. According to the alarmed status, each node sends a data packet including binary data 0 or 1 to the sink. Using the binary data, the sink constructs the likelihood matrix and estimates all the event location. Since binary data is transmitted from local sensors to the processing node, the method needs lower communication energy and less calculation. However, node faults, e.g., false negative, false positive, have a great impact on accuracy of event localization.

Trust and reputation models have been used in the realm of network security [25–28] to detect misbehaving nodes and exclude them from the network. The concept of trust is interpreted as a relation among entities that participate in collaborative protocol in the sensor network system. Trust relations are based on evidence created by the previous interactions of entities within a protocol. Srinivasan [25] proposed a reputation based scheme for excluding malicious beacon node that provide false location information. Probst [27] presents a distributed approach that establishes reputation-based trust among sensor nodes in order to identify malfunctioning and malicious sensor nodes and minimize their impact on applications. In [28], trust is used to indicate the fidelity of event nodes reported in the context of sensor data gathering. It proposes a fault tolerant method to diagnose and mask arbitrary node failures in an event-driven wireless sensor network.

In this paper, we use the trust index model to evaluate the fidelity of information that sensors nodes have reported in the context of multiple event source localization. As the sensor network system runs over a period of time, a number of trust index states are built up as the indicator of the fidelity of data nodes reporting. Then, we reduce the weight of the faulty nodes according to the nodes’ trust index in the process of multiple event location estimation to achieve better fault tolerance performance.

## 3. Model and Assumptions

### 3.1. Assumptions and Definitions

For the sensor network that estimates the position of multiple events, we make the following assumptions:

1. A set of sensor nodes, denoted as *N*, are uniformly spread in the rectangular area *A*. The nodes are static, and their positions are known, denoted as (*x_n_*, *y_n_*), *n* = 1, …, *N*.
2. A set of event sources, denoted as *K*, are randomly distributed at the rectangular area *A*. we assume that the distance between any two event sources is far enough away, and they are not interfering with each other.
3. The event sources emit continuous signals that propagate evenly in all directions.

We assume that the signal strength of the event source *k* (*k* ∈ *K*) is *c_k_*. In addition, the signal strength that the sensor node *n* inspected, denoted as *s_n,k_*, is inversely proportional to the power *α* (*α* ∈ *R^+^*) of distance *r_n,k_* from the sensor node *n* to the event source *k*, in which *α* depends on the environment factor. So we have *s_n,k_* at *t*-th sampling as follows:

(3) $$s_{n,k}(t)=\frac{c_{k}}{r_{n,k}^{\alpha }(t)}$$

where *r_n,k_*(*t*) is the distance of sensor node *n* to source *k* at time *t*, given by:

(4) $$r_{n,k}(t)=\sqrt{{\left(x_{n}-x_{s,k}(t)\right)}^{2}+{\left(y_{n}-y_{s,k}(t)\right)}^{2}}$$

As a result, the *t*-th sampling measurement of a sensor *n* located at (*x_n_*, *y_n_*) is given by the sum of the signal strength from all sources at the sensor location:

(5) $$Z_{n}(t)=\text{min}\{V_{\text{max}},\gamma \sum_{k=1}^{K}s_{n,k}(t)+\omega_{n}(t)\},\;\;n=1,2,\ldots ,N,\;\;\;t=1,2,\ldots M$$

where *V*_max_ reflects the maximum extent of sensor measurement, *γ* is the factor of the sensor gaining ratio. We assume that the signal noise *ω_n_*(*t*) is satisfied with the model of white Gaussian noise, $\omega_{n}(t)∼N(0,\sigma_{\omega }^{2})$, *n* = 1, 2, …, *N*, *t* = 1, 2, …, *M.* Model Equation (5) is commonly used in wireless sensor networks as a signal propagation model [29,30].

We assume that the sensor nodes have been preset with a common threshold *T* of signal strength. The definitions of alarm sensor and non-alarm sensor are given as follows:

- *Alarmed Sensor*: a sensor whose signal measurement value satisfies *Z_n,t_* ≥ *T*.
- *Non-alarmed Sensor*: a sensor whose signal measurement value satisfies *Z_n,t_* < *T*.

Next, we explain some definitions [16,17] which are used in this paper:

**Definition 1:** *ROI* (Region of Influence) is the area around an event source; when a sensor node is located inside this area, it will alarm with high probability.

**Figure 1.**
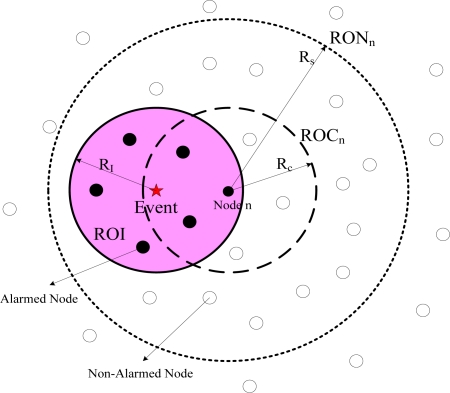
The scenario of various regions used in this paper.

As referred in Equation (3), the *ROI* of a single source is a circle centered at the source location with radius $R_{I}=\sqrt[{\alpha }]{c/T}$ (demonstrated in Figure 1). For multiple sources, the shape and size of the *ROI* depends on the distances between the sources. For any two sources, the *ROI* is connected if and only if their distance *d* ≤ *L* [17], where:

(6) $$L=\frac{1}{\sqrt[{\alpha }]{T}}{\left(\sqrt[{\alpha +1}]{c_{1}}+\sqrt[{\alpha +1}]{c_{2}}\right)}^{\frac{\alpha +1}{\alpha }}$$

If the two event sources are identical, *i.e.*, *c*_1_ = *c*_2_ = *c*, then:

(7) $$L=2\sqrt[{\alpha }]{2}\sqrt[{\alpha }]{\frac{c}{T}}=2\sqrt[{\alpha }]{2}R_{I}$$

where $R_{I}=\sqrt[{\alpha }]{c/T}$ is the radius of the *ROI* of a single event source.

We assume that the distance between any two events is greater than *L*. That is, their *ROI* are not connected.

From the sensor node perspective, we define two more regions for the single source case.

**Definition 2:** *ROC* (Region of Coverage) of sensor node *n* is the area around a sensor node, in which if a event source is located inside, then it will be detected with high probability (as illustrated in Figure 1).

For a single event source, it can be obtained by the expression of Equation (3) that, for a sensor node *n*, *ROC_n_* is an circle area centered at the alarmed sensor node *n*, and is equal to the area of *ROI*, $R_{c}=R_{I}=\sqrt[{\alpha }]{c_{k}/T}$. For multiple event sources, the size of *ROC* is determined by the signal strength of all event inspected by the sensor node. Because we assume that the distance between two event sources is large enough, the strength of distant event source is negligible compared with nearby events.

**Definition 3:** *RON* (Region Of Neighbor) of sensor node is the area around a sensor node, in which the reporting data of all nodes located inside are collected for construction of likelihood function to achieve the estimation of event source location (as shown in Figure 1).

Since energy efficiency is the major issue in sensor networks and communication is the most expensive operation in terms of energy. We assume *RON_n_* = 2*ROC_n_*, which is determined in tradeoff between estimation accuracy and complexity.

### 3.2. Fault Model

We consider two types of node alarm fault in the paper:

- *False positive*: some sensor nodes located outside the *ROI* of the event source are alarmed.
- *False negative*: some sensor nodes located inside the *ROI* of the event source are not alarmed.

This fault model is reflecting two fault types in event localization using binary data which is proposed in SNAP [16]. Due to noise, energy depletion, harsh environmental conditions, sensor malfunction, and so on, sensor nodes may often provide erroneous or unpredictable sensor data which leads to false positive alarms or false negative alarms in event localization using binary data. We introduce this fault model in the event localization of multiple sources in this paper.

### 3.3. Trust Index Model

We are introducing a *trust index* to evaluate the correctness of the observation value of the sensor nodes in the process of event localization, and distinguish the correct nodes, which have high probability of reporting correct data, from faulty nodes. So we treat the data from the correct nodes with higher weight and the data from faulty nodes with lower weight in the maximum likelihood construction for event location estimation, to reduce the influence of faulty nodes on the accuracy of event localization in sensor network.

Each node in the field is assigned a *trust index* (referred to as *TI*, and *TI* ∈ [0, 1]). The trust index of a node is a measurement of the fidelity of event report of that node. The higher the trust index of a node is, the more reliable the node is deemed by the sink. At the initialization of the sensor network system, each node’s trust index is set to 1. The *TI* of node *k* in the *t*-th sampling measurement is defined as:

(8) $$TI_{k,t}=e^{-\lambda \nu_{t}}$$

where *v_t_* is a step variable which is used to control the modification of the trust index value of node *k* in the *t*-th sampling measurement: *TI_k,t_*; *λ* is a constant that decides how fast the *TI_k,t_* will be changed when *v_t_* increases or decreases.

**Figure 2.**
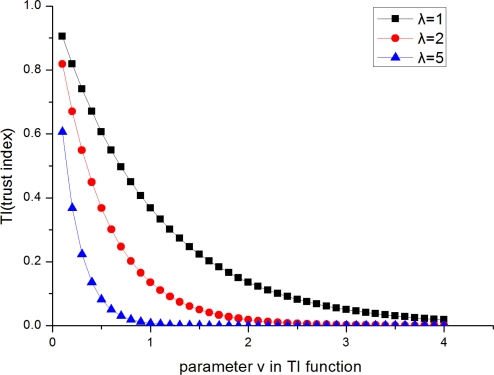
The family curves of *TI.*

Figure 2 depicts the variation of *TI* as the constant *λ* changes. The bigger constant *λ* is, the more dramatically *TI* decreases as the step variable *υ* increases. For a faulty node, it’s better to decrease its *TI* quickly so that it will have less influence on location estimation. However, some new modification errors may be introduced in the process of trust index modification, for example, the trust index of a correct node may be decreased due to wrong alarm. In order to reduce the location errors caused by modification errors, *λ* should be set to a proper value. Therefore, we should make a tradeoff between these two aspects. In the paper an empiric value *λ* = 2 is determined.

As mentioned above, each node’s *TI* is initialized to 1, that is, *υ* is initialized to 0. Similar to the above analysis, the changing step on *υ* has to be a proper value. In the paper, we decide a changing step equal to 0.1. In other words, each time a node makes a report deemed faulty by the sink, its *TI* value is increased by a step 0.1. On the contrary, each time a node makes a report deemed to be correct by the sink, its *TI* value is decreased by a step 0.1 if *υ* is larger than 0. The rules for modification of *TI* are given as follows:

(9) $$\nu_{t+1}=\{\begin{array}{ll} 0 & the\;node\;is\;deemed\;as\;normal\\ \nu_{t}-0.1 & the\;node\;is\;deemed\;as\;normal\;and\;\nu_{t}>=0.1\\ \nu_{t}+0.1 & the\;node\;is\;deemed\;as\;faulty \end{array}$$

## 4. TISNAP Algorithm

In this section, we introduce the Trust Index based Subtract on Negative Add on Positive (TISNAP) localization algorithm, which reduces the impact of faulty nodes on the event localization by decreasing their trust index, to improve the accuracy of event localization and performance of fault tolerance for multiple event sources localization. It has three phases:

### 4.1. Identifying the Number of Events

In multiple events localization, the first step is to identify the number of events in an area, and this is the precondition for estimating the location of the event sources. During the phase, the alarmed nodes send ‘1’ (alarm packet) to the sink, other nodes remain silent. In the sampling period, if the sink did not receive the alarm packet from a node, the sink regards it as a non-alarmed node. After the sink collected all alarm data in a sampling period, it computes the following likelihood function *F_n_* for a sensor node *n* using information from the neighboring nodes that is located inside *ROC_n_* of node *n*:

(10) $$F_{n}=\sum_{m\in ROC_{n}}b_{m}$$

where:

(11) $$b_{m}=\{\begin{array}{l} +1\times TI_{m},\;\;\text{node }m\text{ is alarmed}\\ -1\times TI_{m},\;\;\text{otherwise} \end{array}$$

This process is equivalent to the majority voting rule. By introducing the trust index of nodes, the algorithm enhances the influence of normal nodes and reduces the influence of faulty nodes in the likelihood function. Then the sink selects the alarmed nodes, whose corresponding likelihood function values are the maximal value in their surrounding area respectively, as the cluster nodes. Generally, the number of cluster nodes is equal to the number of event sources which we can find in the whole area. The algorithm of selecting cluster nodes is shown in Algorithm 1:

**Algorithm 1.** Finding the cluster nodes.

**Input:** [X_n_, Y_n_, F_n_]for sensor nodes n = 1,2, …, N which F_n_> 0 **Output:**[X_m_, Y_m_] for sensor nodes m = 1,2,…, M which M < N 1:**for all** sensor nodes i = 1,2,…, N 2: **for all** sensor nodes j = 1,2,…, K ∈ ROC_i_ 3: **if** F_j_> F_i_ 4: **break**; 5: **else** 6: count++; 7: **end for** 8: **if** count == K //F_i_ is larger than all F_j_ which j = 1,2,…, K 9: **output:**[X_i_, Y_i_] // cluster nodes 10: **end for**

### 4.2. Event Localization

This phase is mainly used to estimate the location of all event sources. We divide the phase into three steps:

#### 4.2.1. Grid Formation

**Figure 3.**
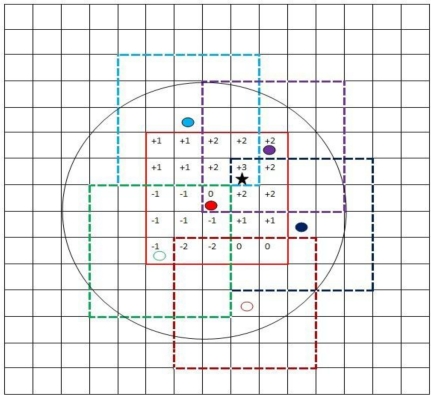
Likelihood matrix *L* calculated by the sink.

The area is divided into a grid with *G* × *G* cells and grid resolution *l*, e.g., Figure 3 shows a 30 × 30 field with *G* = 15 and a grid resolution *l* = 2. Let *C*(*i*, *j*) for *i*, *j* = 1, …, *G*, denote the centers of these cells in a matrix. The number of cells is a trade-off between estimation accuracy and complexity. Each sensor node is associated with a cell(*i*, *j*) based on its position (depending on the resolution, a cell may contain multiple sensors or no sensor at all). The position index of each node is denoted by (*X_n_*, *Y_n_*), *n* = 1, …, *N*, where *X_n_*, *Y_n_* ∈ {1, 2, …, *G*}.

#### 4.2.2. Construction of the Likelihood Matrix

Since the events are highly likely to occur in the *ROC* of the cluster node, for a cluster node *k*, we define a matrix *L_k_*. Using the information from all relevant sensor nodes inside the *RON_k_* of the cluster node *k*, the sink constructs a corresponding likelihood matrix *L_k_*.

The cluster node *k* is associated with *g_k_*, a sub-grid with G_k_ × G_k_ cells, centered around its location (*X_k_*, *Y_k_*). The size of the sub-grid *G_k_* depends on the size of the *RON_k_* and the grid resolution *l*:

(12) $$G_{k}=\left\lfloor \frac{2R_{k}}{l}\right\rfloor +1$$

The sink defines a *G_k_* × *G_k_* likelihood matrix *L_k_* where each element (*i*, *j*) of *L_k_* corresponds to a cell (*u*, *v*) of *g_k_*. The relation is given by a mapping *M*: *g_k_* → *L_k_* :

(13) $$M({\left[u,v\right]}^{T})={\left[u-X_{k}+\left\lceil \frac{G_{k}}{2}\right\rceil ,v-Y_{k}+\left\lceil \frac{G_{k}}{2}\right\rceil \right]}^{T}$$

where *u*, *v* ∈ {1, 2, …, *G*}. For every element of *L_k_*, the sink adds the contribution of each sensor that has the corresponding cell in *ROC* of the cluster node *k*. The contributions depend on the sensor’s state: “+” the trust index of the senor on alarmed and “−” the trust index of the sensor on non-alarmed. More specifically, the sink updates every element (*i*, *j*) of *L_k_* using:

(14) $$L_{k}(i,j)=\sum_{m\in RON_{k}}b_{m}(i,j),i,\;j\in \{1,2,\ldots ,G_{k}\}$$

where:

(15) $$b_{m}(i,j)=\{\begin{array}{l} +1\times TI_{m}\;\text{if node }m\;\text{alarmed and }M^{-1}(i,j)\in ROC_{m}\\ -1\times TI_{m}\;\text{if node }m\;\text{non}-\text{alarmed and }M^{-1}(i,j)\in ROC_{m}\\ 0 \end{array}$$

and *ROC_m_* is the set of all grid cells that are covered by the *ROC* of sensor node *m.* The algorithm of constructing the likelihood function is shown in Algorithm 2:

**Algorithm 2.** Likelihood Matrix Construction.

**Input:** [*X_n_*, *Y_n_*, *b_n_*] for sensor nodes *n* = *1*, *2*, …, *N_k_* ∈ *RON_k_* **Output:** Likelihood matrix *L_k_* 1: *L* ← 0 // initialization 2: **for all** cells *M^−1^(i*, *j)* ∈ *g_k_***do** 3: **for all** sensor nodes *n* that have cell *M^−1^(i, j)* ∈ *ROC_n_***do** 4: *L_k_(i, j)* ← *L_k_(i, j)* + *b_n_*; 5: **end for** 6: **end for**

#### 4.2.3. Maximization

Let (i*, j*) be the element of *L_k_* with the maximum value, *i.e*., *L_k_*(i*, j*) ≥ *L_k_*(*i*, *j*), ∀*i*, *j* = 1, …, *G_k_*. Then *C*(i*, j*) is regarded as one of the location of the events. The center of the cell corresponding to the maximum value of each matrix is regarded as the location of the events. In cases where more than one elements of a matrix have the same maximum value, the estimated event position is the centroid of the corresponding cell centers.

#### 4.2.4. Example

**Figure 4.**
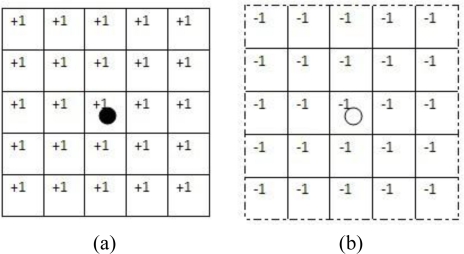
the *ROC* of sensor nodes (**a**) alarmed nodes (**b**) non-alarmed nodes.

We provide a simple example to illustrate the TISNAP algorithm. In the example, the *ROC* of sensor node *n* is the set of cells that fall in a square of 5 × 5 cells around cell (*i*, *j*), where sensor *n* is located, as shown in Figure 4. The *TI* of each node is 1.

Figure 3 demonstrates the algorithm used by the sink for constructing the likelihood matrix *L_k_* corresponding to node *k*. In Figure 3, the red node is the cluster node and there are three alarmed nodes and two non-alarmed nodes in its *RON*. Using the information from all relevant sensor nodes inside the *RON* of the cluster node, the sink constructs the likelihood matrix. The maximum value of the matrix is 3 and the center of the cell corresponding to it is the location of the event we estimated.

### 4.3. Updating the Trust Index

**Figure 5.**
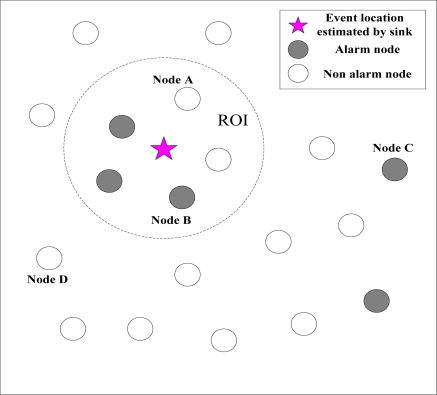
The state of nodes located in different regions.

According to the estimated location of the events, the sink decides whether all the information reported by nodes is true or false after a round of event localization operation. Then, the sink updates the *TI* of all nodes according to Equations (8) and (9). If the node is deemed as normal, the sink will increase its *TI*. Otherwise, the sink will reduce it. To illustrate the case, we provide a simple example. We assume that using the event localization algorithm, the location of the event estimated by the sink is shown in Figure 5 in the *t*-th sampling period. Then, based on the estimated location of the event, the sink updates the trust index of all nodes for the preparation of the next round of event localization operation. As Figure 5 shows, updating the trust index of the node has the following situations:

- The node is in the *ROI* of the event, but it is not alarmed. The sink considers it as *false negative* node and reduces its *TI* value. Just as node A in Figure 5, according to Equations (8) and (9), the trust index is given by:
  $$\nu_{A,t+1}=\nu_{A,t}+0.1,\;\;TI_{A,t+1}=e^{-\lambda \nu_{A,t+1}}$$
  So the *TI* of node A is reduced.
- The node is in the *ROI* of the event, and it is alarmed. The sink considers it *normal* node and increases its trust index. Just as node B in Figure 5, the trust index is given by:
  $$\nu_{B,t+1}=\nu_{B,t}-0.1,TI_{B,t+1}=e^{-\lambda \nu_{B,t+1}}$$
  So the trust index of node B is increased.
- The node is out of the *ROI* of the event, but it is alarmed. The sink considers it *false positive* node and reduces its trust index. Just as node C in Figure 5, the trust index is given by:
  $$\nu_{C,t+1}=\nu_{C,t}+0.1,\;TI_{C,t+1}=e^{-\lambda \nu_{C,t+1}}$$
  So the trust index of node C is reduced.
- The node is out of the *ROI* of the event, and it is not alarmed. The sink considers it normal and increases its trust index. Just as node D in Figure 5, the trust index is given by:
  $$\nu_{D,t+1}=\nu_{D,t}+0.1,\;TI_{D,t+1}=e^{-\lambda \nu_{D,t+1}}$$
  So the trust index of node D is increased.

## 5. Theoretical Analysis

In this section, we theoretically compare the TISNAP algorithm with the DSNAP one. The DSNAP algorithm is similar to the SNAP algorithm in [16], and in essence, they are all methods of maximum likelihood estimation which use the information of sensor nodes located in the area of event source’s *ROI*. DSNAP is used for multiple event sources localization, while SNAP is used for single event source localization. According to the description of the literature [16], we assume that a set of sensor nodes, *K*, located in an event source’s *ROI* area. For node *k*, *k* ∈ *K*, we define the indicator function *I_k_* for *k* = 1, …, *K* and *t* = 1, …, *M*:

(16) $$I_{k,t}=\{\begin{array}{ll} 0, & {\text{Z}}_{\text{k},\text{t}}<T\\ 1, & {\text{Z}}_{\text{k},\text{t}}\geq T \end{array}$$

Thus, the sensor data can be represented as *I* = {*I_k,t_*: *k* = 1, …, *K*, *t* = 1, …, *M*}. The goal is to estimate the source location *θ* = [*x_k_*, *y_k_*] using the collected data *I*. The joint likelihood function is given by:

(17) $$\text{lg}p(I_{k}|\theta )=\sum_{k=1}^{K}\sum_{t=1}^{M}I_{k,t}\times \text{lg}[Q(\frac{T-S_{k}(\theta )}{\sigma_{\omega }})]+(1-I_{k,t})\times \text{lg}[1-Q(\frac{T-S_{k}(\theta )}{\sigma_{\omega }})]$$

where $Q(x)=\frac{1}{\sqrt{2\pi }}\int_{x}^{\infty }e^{-\frac{t^{2}}{2}}dt$ and *S_k_*(*θ*) is the signal that would have been measured by sensor *k* if the source was at location *θ* and there was no noise (given by Equation (5)). In [16], they propose the following arbitrary probability assignment for their indicator function *I_k,t_*:

$$\text{Pr}\{I_{k,t}=1|\theta \}=Q(\frac{T-s_{k}(\theta )}{\sigma_{\omega }})=0.99,$$

$$\text{Pr}\{I_{k,t}=0|\theta \}=1-Q(\frac{T-s_{k}(\theta )}{\sigma_{\omega }})=0.01$$

Next, consider the modified likelihood function *p′*(*I_k_* | *θ*) = 10^2*KM*^ *p*(*I_k_* | *θ*). Taking the logarithm of the modified likelihood function, they get:

(18) $$\begin{array}{ll} \text{log}p\prime (I_{k}|\theta ) & =\sum_{k=1}^{K}\sum_{t=1}^{M}I_{k,t}\times \text{log}(9.9)+(1-I_{k,t})\times \text{log}(0.1)\\ & \approx \sum_{k=1}^{K}\sum_{t=1}^{M}I_{k,t}\times (+1)+(1-I_{k,t})\times (-1) \end{array}$$

The SNAP estimator is given as the following:

(19) $${\hat{\theta }}_{SNAP}=\underset{\theta }{\text{max}}\text{log}p\prime (I_{k}|\theta )$$

When constructing the likelihood function, the TISNAP algorithm has taken into account the impact of faulty nodes. The sink assigns a trust index to every node, and the impact of faulty nodes is reduced. Therefore the algorithm has better performance of fault tolerance. Based on Equation (18), the joint likelihood function we define is given by:

(20) $$\text{log}p^{\prime \prime }(I_{k}|\theta )=\sum_{k=1}^{K}\sum_{t=1}^{M}TI_{k,t}[I_{k,t}\times (+1)+(1-I_{k,t})\times (-1)]$$

where:

(21) $$TI_{k,t}=e^{-\lambda \nu_{t}}$$

and *TI_k,t_* denotes the trust index of node *k* in the *t*-th sampling period. *v_t_* is obtained by Equation (8).

Based on the Equation (18), *F_k,t_* denotes the impact on the likelihood function by node *k* in the *t*-th sampling period. It is given by:

(22) $$F_{k,t}=I_{k,t}\times (+1)+(1-I_{k,t})\times (-1)$$

If the node is alarmed, *I_k,t_* = 1 is obtained by Equation (16). Then *F_k,t_* = 1. Otherwise, *I_k,t_* = 0 and *F_k,t_* = −1. However, when the node is faulty, the alarm status of the node is the opposite. A node should have been alarmed under normal conditions, but it is non-alarmed due to a fault, so ${I^{\prime }}_{k,t}=0$ is obtained by Equation (16) and ${F^{\prime }}_{k,t}=-1$. Similarly, a node should have been non-alarmed under normal conditions, but it is alarmed due to a fault, so ${I^{\prime }}_{k,t}=1$ and ${F^{\prime }}_{k,t}=1$. In the DSNAP algorithm, the difference caused by a single faulty node is 2. Therefore, with the increasing of faulty nodes, the likelihood function will be greatly affected.

However, in this paper, the sink assigns a trust index to every node and the impact of faulty nodes is reduced. Based on the Equation (20), *FI_k,t_* denotes the impact on the likelihood function by node *k* in the *t*-th sampling period. It is given by:

(23) $$FI_{k,t}=TI_{k,t}[I_{k,t}\times (+1)+(1-I_{k,t})\times (-1)]$$

According to the Equation (9), if the node is normal, its trust index is 1, so *FI_k,t_* = *F_k,t_*. However, when the node is faulty, the alarm status of the node is the opposite and the difference caused by a single faulty node is 2*TI_k,t_*. According to the Equations (8) and (9), after several rounds of event localization operations, the *TI_k,t_* of the faulty node *k* is greatly reduced after *t*-th sampling period and it plays a minimal role in the process of event localization. Therefore, in the TISNAP algorithm, the value of the likelihood function is mainly determined by the normal nodes. The algorithm reduces or even ignores the impact of the faulty nodes. It is the reason that the TISNAP algorithm has better fault-tolerant performance and higher accuracy of localization after several rounds of event source locating operations.

## 6. Performance Evaluation

All experiments in this paper are performed in a simulation environment. In the experiments, we use a square 200 × 200 sensor field with *N* = 1,000 randomly deployed nodes. We assume that the nodes in the sensor network gradually become faulty nodes over time. In the beginning, all nodes are normal. As time goes through, the number of faulty nodes increases at the rate of 5%. Two event sources are randomly deployed in the area and their distance is not less than $2\sqrt{2}ROC$. The signal strength at the location of the sources is identical. For the parameters used in the experiments, we use the default values shown in Table 1. According to Equation (5), the sensor readings are given by:

(24) $$Z_{n}(t)=\text{min}\{3000,\sum_{k=1}^{K}\frac{c_{k}}{r_{n,k}^{2}(t)}+\omega_{n}(t)\}$$

We use the root mean square error (RMS Error) as a method of performance evaluation. We assume that the actual location of the two event sources is $(x_{s,b}^{1},y_{s,b}^{1})\in A$ and $(x_{s,b}^{2},y_{s,b}^{2})\in A$. The location of the two event sources estimated by TISNAP algorithm is ( ${\hat{x}}_{s,b}^{1}$, ${\hat{y}}_{s,b}^{2}$) and ( ${\hat{x}}_{s,b}^{1}$, ${\hat{y}}_{s,b}^{2}$), where *b* = 1, …, *B*. The RMS Error is given by:

(25) $$RMS\;Error=\frac{1}{2B}\sum_{k=1}^{B}\left(\sqrt{{\left(x_{s,k}^{1}-{\hat{x}}_{s,b}^{1}\right)}^{2}+{\left(y_{s,k}^{1}-{\hat{y}}_{s,b}^{1}\right)}^{2}}+\sqrt{{\left(x_{s,k}^{2}-{\hat{x}}_{s,b}^{2}\right)}^{2}+{\left(y_{s,k}^{2}-{\hat{y}}_{s,b}^{2}\right)}^{2}}\right)$$

In this paper, we assume that B = 100. In every experiment, the location of the sensor nodes is fixed and the event sources are randomly deployed in the area.

### 6.1. Fault Tolerance

**Figure 6.**
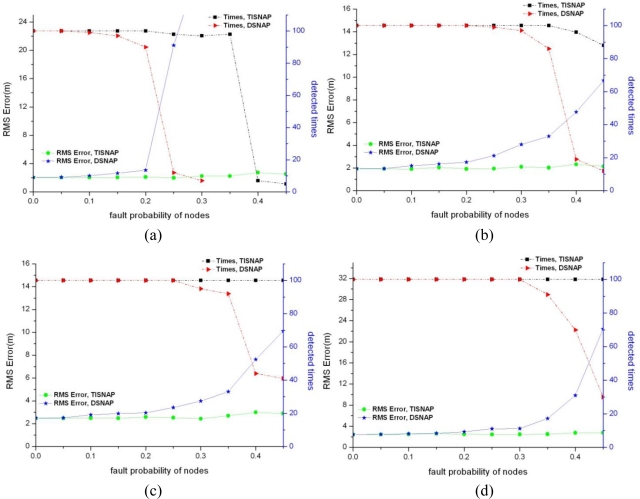
Fault tolerance performance for different signal strength of event sources. (**a**) c = 1,000; (**b**) c = 2,000; (**c**) c = 3,000; (**d**) c = 4,000.

In this section, we evaluate the performance of fault tolerance of the TISNAP algorithm and the DSNAP algorithm under conditions of different fault probability and different numbers of alarmed sensor nodes. Also, we observe how many times all the event sources can be detected in 100 tries and how much the location deviation is. We assume that there are two fault types in the area: one is a *false negativ*e, that is, sensor nodes that fall inside the *ROI* of the event source but their observed readings are smaller than threshold *T*, so they are not alarmed. The other is a *false positive*, that is, sensor nodes that fall outside the *ROI* of the event but their observed readings (we assume the observed reading is a random value between threshold *T* and the source signal strength *c*) are greater than threshold *T*, so they are alarmed. Four groups of experiments are performed under different signal strength of event source, as shown in Figure 6. Left y-axis denotes RMS Error and right y-axis denotes the times all the events are detected in 100 experiments.

As shown in Figure 6, the TISNAP algorithm has better fault tolerance performance than the DSNAP algorithm. When the fault probability of nodes is higher than 35%, the number of event sources in the area cannot be accurately identified using the DSNAP algorithm. When the fault probability of nodes is 40%, the times of that all event sources are detected is less than 40% in 100 tries. However, in the TISNAP algorithm experiment, when the fault probability of nodes is less than 50%, 100% of event sources can be accurately detected and the RMS error is smaller. In the TISNAP algorithm, because the sink assigns a trust index to every node, the trust index of most faulty nodes is reduced after several times of localization, and the trust index of most normal nodes remains high. Hence, the data of the normal sensor nodes have more weight in the process of event source localization. Therefore, the TISNAP algorithm has higher accuracy of localization.

### 6.2. Dropped Packets

**Figure 7.**
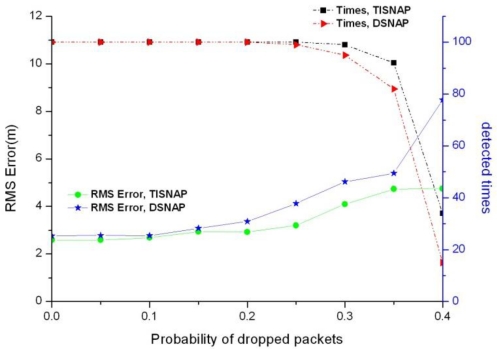
The Fault tolerance performance under different probability of dropped packets.

In this section, we investigate the performance of the two algorithms if packets are dropped by the network. As mentioned in Section 4.1, in the first phase of TISNAP, each alarmed node sends a data packet to the sink and other nodes remain silent. Therefore, in the sampling period, if the sink does not receive the packet from a node, it will regard it as a non-alarmed node and assumes that the node does not detect the events. To investigate the effect of dropped packets, we assume that there is only one kind of fault which is dropping packets. And each node has the same probability of dropping packets. Figure 7 shows the impact of dropped packets on the two algorithms.

As shown in Figure 7, under the same packet loss rate, the TISNAP algorithm has higher positioning accuracy and better performance of fault tolerance than the DSNAP one. When the packet loss rate is higher than 35%, neither of them cannot accurately determine the number of the events in the area, because the packet loss rate of nodes has a great influence on the alarmed nodes and the alarmed nodes play an important role in the process of event localization. However, non-alarmed nodes do not need to send packets to the sink, so packet loss rate has no impact on them.

### 6.3. Board Overheating

**Figure 8.**
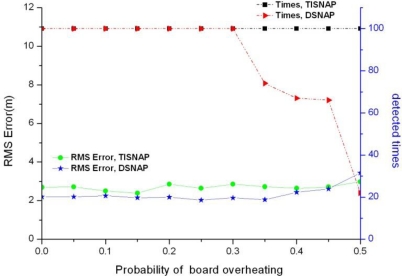
Estimator performance *versus* probability of overheating.

In sensor networks, due to working long hours, the boards of sensor nodes may be overheating and this may cause the sensor nodes to report false events, as the node is always alarmed. We assume that each node has the same probability of the fault of board overheating. Figure 8 shows the impact of board overheating on the two algorithms.

As shown in Figure 8, the TISNAP algorithm we proposed has better fault tolerance performance to board overheating. When the probability of fault is 50%, it still can accurately determine the number of events in the area and estimate their location. However, when the probability of fault is larger than 30%, the performance of the DSNAP algorithm begins to decline sharply, and when the fault probability is 40%, the number of times all events are detected is less than 70% in 100 tries.

## 7. Conclusions

TISNAP is a simple, efficient, fault-tolerant localization algorithm for multiple event source localization in sensor networks. It only uses the binary data reporting from the sensor nodes in the localization process. The trust index model is introduced to measure the fidelity of data reported by sensor node and to reduce the impact of faulty nodes on the multiple event localization by decreasing their trust index value. Compared to the DSNAP, TISNAP has the same computational overhead but can achieve higher accuracy in multiple event localization when a large percentage of the sensor nodes report erroneous observations. Experimental results show that when 50% nodes are in failure mode, the algorithm can still identify all events correctly and accurately estimate their location. For our future work, we plan to study the performance of TISNAP with respect to energy, bandwidth, and QoS. Furthermore, we will investigate real propagation models, such as in problems of environmental pollution, where an actual substance is released in the environment. Finally, we try to combine this algorithm with Kalman Filtering to achieve tracking of multiple event sources.

## Figures and Tables

**Table 1.** Default Parameter Values.

**Parameter**	**Symbol**	**Default Value**
The area	A	200 m × 200 m
Number of sensor nodes	*N*	1,000
Saturation voltage	*V_max_*	3,000
Source amplitude	*c*	3,000
Noise variance	*ω_n,t_*	*ω_n,t_* ∼ *N*(0,1)
Threshold	*T*	14
Grid resolution	*g*	1
Scaling factor	*α*	2
Sensor gain	*γ*	1
